# Delayed anaphylaxis due to Alpha-gal allergy: A modified desensitization protocol with red meat in an adult patient

**DOI:** 10.5578/tt.20239714

**Published:** 2023-09-22

**Authors:** M.E. Tepetam, Z. Yegin Katran, R. Bayraktar Barın, B. Çakmak Uğurlu

**Affiliations:** 1 Clinic of Immunology and Allergy, University of Health Sciences Süreyyapaşa Chest Diseases and Thoracic Surgery Training and Research Hospital, İstanbul, Türkiye; 2 Clinic of Pulmonology, University of Health Sciences, Süreyyapaşa Chest Diseases and Thoracic Surgery Training and Research Hospital, İstanbul, Türkiye

**Keywords:** Delayed anaphylaxis, Alpha-gal allergy, red meat desensitization

## Abstract

**ABSTRACT:**

**Delayed anaphylaxis due to Alpha-gal allergy: A modified desensitization protocol with red meat in an adult patient:**

Alpha-gal allergy is the sensitization to Alpha-gal present in saliva when a tick bites and the development of an IgE-mediated reaction to Alpha-gal also present in red meat by cross-reactivity. In contrast to other food allergies, symptoms occur as late as 2-6 hours after a meal. Prick to prick testing with nonmammalian meat in combination with cooked mammalian meat is recommended for diagnosis. However, the main diagnostic test is Alpha-gal sIgE> 0.1 IU/mL. The primary recommendation in patients with Alpha-gal syndrome is to prevent new tick bites and avoid all mammalian meats. Since most of the dishes in our country’s food culture contain red meat, elimination diet may adversely affect patients quality of life. In the management of these patients, the option of desensitization with red meat can be considered by evaluating the benefit-risk ratio together with the patient. Our patient with a history of tick bites and a reaction pattern ranging from urticaria to anaphylaxis two hours after meat consumption was evaluated for Alpha gal allergy. The patient was found to be positive by prick-to-prick with cooked red meat. In addition, the high level of Alpha-gal specific IgE (27.3 Ku/L) confirmed the Alpha-gal allergy, and the decision to apply desensitization with red meat was taken. There are only two literatures on this subject, one of which includes two adult cases and the other a single pediatric case. Since a reaction developed in the fifth step of the 27-step desensitization scheme (Ünal et al.), which we took as a reference, which led to a dose increase of more than 100 times, we modified the protocol by using an intermediate steps. We repeated the prick-to-prick test with red meat after desensitization in our case who successfully completed the modified desensitization protocol. Observation of more than half reduction in test edema diameter concretely supports the success of our modified desensitization protocol.

## Abstract (tr)

**Alfa-gal alerjisine bağlı gecikmiş anafilaksi: Yetişkin bir hastada kırmızı et ile modifiye desensitizasyon protokolü:**

Alfa-gal alerjisi, bir kene ısırdığında tükürükte bulunan Alfa-gal’a karşı duyarlanma oluşması ve çapraz reaktivite ile kırmızı ette de bulunan Alfa-gal’a karşı IgE aracılı bir reaksiyon gelişmesidir. Diğer gıda alerjilerinin aksine, belirtiler yemekten 2-6 saat kadar sonra ortaya çıkar. Tanı için pişmiş memeli eti ile birlikte memeli olmayan et ile prick-to-prick testi önerilir. Bununla birlikte, ana tanı testi Alfa-gal sIgE> 0,1 IU/mL’dir. Alfa-gal sendromlu hastalarda öncelikli öneri yeni kene ısırıklarının önlenmesi ve tüm memeli etlerinden kaçınılmasıdır. Ülkemiz yemek kültüründe yer alan yemeklerin çoğu kırmızı et içerdiğinden eliminasyon diyeti hastaların yaşam kalitesini olumsuz etkileyebilir. Bu durumdaki hastaların yönetiminde, yarar risk oranı hastayla birlikte değerlendirilerek kırmızı et ile desensitizasyon seçeneği düşünülebilir. Kene ısırığı öyküsü olan ve et tüketiminden iki saat sonra ürtikerden anafilaksiye kadar değişen reaksiyon paterni gelişen hastamız Alfa-gal alerjisi açısından değerlendirildi. Hastaya pişmiş kırmızı et ile yapılan prick-to-prick ile pozitif bulundu. Ayrıca yüksek Alfa-gal spesifik IgE düzeyi (27,3 Ku/L) ile Alfa-gal alerjisi doğrulandı ve kırmızı et ile desensitizasyon uygulama kararı alındı. Bu konuda biri iki yetişkin, diğeri tek bir pediatrik vakayı içeren sadece iki literatür bulunmaktadır. Referans aldığımız 27 basamaklı desensitizasyon şemasının (Ünal ve ark.) yüz kattan fazla doz artışına yol açan beşinci basamağında reaksiyon geliştiğinden ara basamaklar kullanarak protokolü modifiye ettik. Modifiye edilmiş desensitizasyon protokolünü başarıyla tamamlayan olgumuzda desensitizasyon sonrasında kırmızı et ile prick-to-prick testini tekrarladık. Test ödem çapında yarıdan fazla azalma gözlenmesi, modifiye desensitizasyon protokolümüzün başarısını somut olarak desteklemektedir.

## INTRODUCTION

Although the incidence and prevalence of meat allergies in the general population are unknown, it is relatively rare. Among the patients with food allergy, meat allergy has been reported in approximately three to 15 percent of pediatric cases ( 1 , 2 ) and three percent of adult cases ( 3 ). Although the types of meat causing allergy vary according to geographical differences, beef allergy has been reported most frequently (1.5- 6.5%) among children with atopic dermatitis or food allergy/intolerance ( 4 , 5 ). However, specific IgE (sIgE) against bovine meat and bovine serum albumin (BSA) were evaluated in these studies. In the Alpha-gal syndrome described in 2009, which is mostly seen in adulthood, it was shown that sIgE was produced for galactose-a-1,3-galactose (Alpha-gal), a disaccharide structure expressed on the surface of mammalian glycolipids and glycoprotein ( 6 ). This clinical observation was supported by Chung et al. ( 7 ) who reported in 2008 that Alpha-gal was a potential cause of anaphylactic reactions to cetuximab. In the following years, it was shown that there was a strong positive correlation between the history of being bitten by the tick species Amblyomma americanum and sIgE levels developed against Alpha-gal ( 8 ). Many cases of red meat allergy have been reported from our country, especially from the Eastern Black Sea Region where Ixoides ricinus species ticks are abundant ( 9 , 10 ). Although it is thought that IgE-mediated reaction develops against Alpha-gal, which is also present in red meat, with sensitization and cross-reactivity against Alpha-gal present in saliva when the tick bites, it is predicted that type I allergic sensitization may also occur against molecules in carbohydrate structure other than Alpha-gal ( 11 ).

Since symptoms in Alpha-gal syndrome occur as late as 2-6 hours after a meal, unlike other food allergies, the fact that it is not considered as a trigger delays the diagnosis. The fact that Alpha-gal syndrome presents with findings ranging from pruritus to isolated urticaria, angioedema and anaphylaxis in addition to gastrointestinal symptoms, which are not rare, complicates the management of these patients ( 12 , 13 , 14 ). While skin prick test with exracts of mammalian meat is not reliable in the diagnosis, intradermal test with diluted forms or gelatin can be performed. Prick to prick testing with nonmammalian meat (control) in combination with cooked mammalian meat is recommended as an alternative ( 15 , 16 ). However, the main diagnostic test is an Alpha-gal sIgE> 0.1 IU/mL with 100% sensitivity and 92.3% specificity ( 12 , 17 ). Oral provocation with red meat may be recommended in case of incompatibility between history (tick bite and reaction after red meat consumption) and test (Alpha-gal sIgE positivity) ( 18 ).

The primary recommendation in patients with Alphagal syndrome is the prevention of new tick bites and avoidance of all mammalian meats. It is hypothesized that cooking does not denature the Alpha-gal epitope but may reduce the severity of the reaction due to reduced fat content. Nevertheless, since the probability of reaction cannot be eliminated, it is safe to exclude these staple foods from the diet. In addition to this challenging diet, the fact that foods and drugs containing gelatin, vaccines, heparin, thyroid hormones, pancreatic enzyme extracts and monoclonal antibodies such as cetuximab contain Alpha-gal makes accidental exposure of these patients to Alpha-gal antigen ( 18 ). Elimination diet and fear of accidental exposure to Alpha-gal may adversely affect patients’ quality of life. In the management of these patients, the option of desensitization with red meat can be considered by evaluating the benefit-risk ratio together with the patient. To the best of our knowledge, there are only two reports in the literature on this subject, one of which includes two cases and the other is a single pediatric case ( 10 , 19 ). We performed desensitization after confirmation of Alpha-gal allergy with sIgE in our patient who had a history of tick bites and had a reaction pattern ranging from urticaria to anaphylaxis two hours after meat consumption several times. We used, as an example, the 27-step desensitization scheme of Ünal et al. ( 10 ), which they successfully applied in two adult patients, Since our patient developed a reaction after the fifth step [fifth step= 0.5 mg, fourth step: 40 drops; 0.002 mg (cumulative daily dose= 0.004 mg); approximately 125-fold dose increase from the total daily dose], we modified the protocol using intermediate steps. We wanted to present our patient who developed a reaction in the original protocol and successfully completed the modified desensitization protocol.

## CASE REPORT

A 38-year-old male patient was admitted to our immunology and allergy outpatient clinic with pruritus, urticaria and sometimes chest tightness, wheezing, two hours after eating beef or mutton. The patient did not have any previous known disease. There was no concomitant urticaria, angioedema, asthma, rhinitis or atopic dermatitis. The patient developed urticarial plaques for the first time 15 years ago within two hours after eating boiled red meat. After this event, he stated that the reaction index was aggravated every time he ate meat. Initially, the patient developed only urticaria plaques on the body two hours after eating meat, but when chest pressure, congestion and shortness of breath started to develop over time, he stopped consuming meat completely. The patient, who worked in a hazelnut garden in summer, had been bitten by ticks many times before. He could consume fish, chicken and dairy products without any problems. The patient who wanted to consume meat-containing foods very much applied to our outpatient clinic.

**Figure 1:**
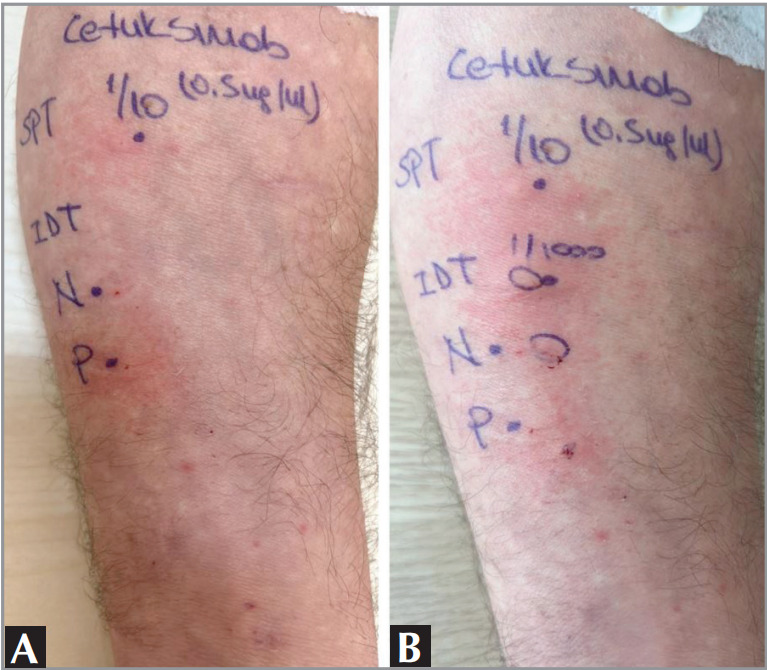
A. Positive prick test (7 x 6 mm) with cetuximab 1/10 dilution (0.5 mg/mL). P: Histamine (as positive control); N: Saline (as negative control); reading at 15 minutes. B. Positive intradermal test (10 x 4 mm) with cetuximab 1/1000 dilution (0.005 mg/mL).

In routine laboratory tests, hemogram and biochemistry parameters were normal and ENA and ANCA profiles were negative. Absolute eosinophil count was 210 cells/μL (2.7%), tryptase= 2.42 Ug/L, serum total IgE 461 IU/mL. No variable airflow obstruction was detected in pulmonary function test. Skin prick test with respiratory allergens including house dust mite, cat, dog dander, mold fungi were negative and food allergen extracts including beef, milk, soy, egg, chicken, banana, hazelnut, peanut were also negative. Serum sIgE: cow’s milk was negative and beef sIgE= 0.160 Ku/L. Prick to prick test with cooked beef was 17 x 12 mm; Alpha-gal (Gal-Alpha-1,3-Gal) sIgE level was 27.3 Ku/L. In the multiparametric assay Allergy Explorer (ALEX) test, a component-based test performed at the patient’s own request; House cricket (house cricket, Acheta domesticus; Ach d)= 2.91 Ku/L, migratory locust (Loc m)= 0.79 Ku/L, mealworm (Ten m)= 1.34 Ku/L and honey bee (Api M1= 1.41 Ku/L, Api m10= 4.64 Ku/L) were sIgE positive. Nearly 300 other components were reported as sIgE negative. The prick test at the recommended nonirritant concentration ( 21 ) with cetuximab was positive (7 x 6 mm) at 1/10 dilution (0.5 mg/mL). Intradermal test starting at 1/1000 dilution (0.005 mg/mL) was also evaluated as positive (10 x 4 mm) ( Figure 1 ).

**Figure 2:**
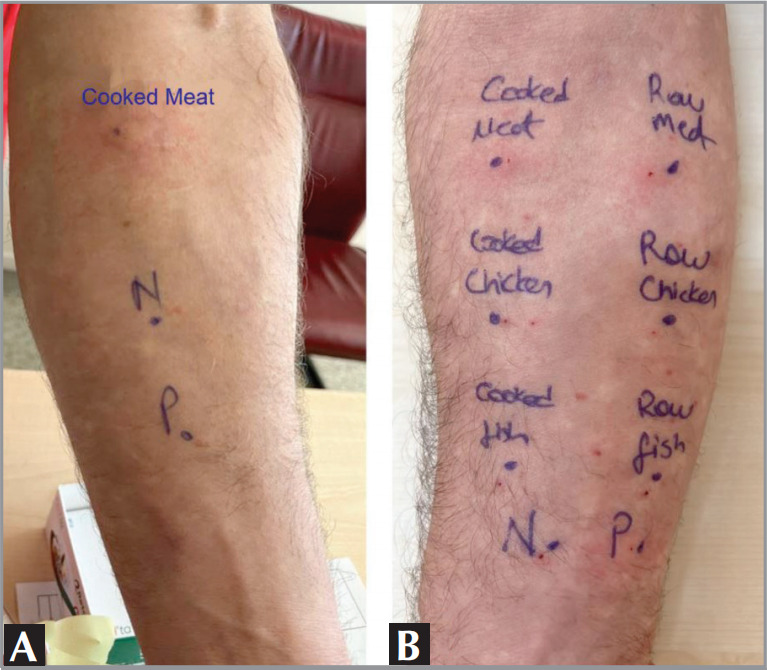
A. Positive prick to prick test with cooked meat (17 x 12 mm, with pseudopod) before desensitization. P: Histamine (as positive control); N: Saline (as negative control); reading at 15 minutes. B. Positive prick to prick test with cooked (3 x 4 mm) and raw meat (3 x 3 mm) but negative with cooked and raw chicken and fish.

The patient with a diagnosis of Alpha-gal allergy and planned desensitization with meat was hospitalized and written informed consent was obtained. Cooked meat extract was started with 10 drops (0.0005 mg) in 1/1000 dilution as recommended by Unal et al. and 10 drops were given again after two hours. When the dose was changed from 40 drops (0.002 mg) to 0.5 mg, local urticaria plaque developed on the anterior abdominal wall and the patient was followed up without treatment. After spontaneous regression of the symptoms, 50 drops (0.003 mg) were given the next day and the step intervals were opened. On the days that coincided with the weekend, the same dose he received on Friday was given. Our desensitization protocol was successfully completed with a final daily dose of 120 mg in 39 steps ( Table 1 ). After desensitization, the prick to prick test repeated with cooked beef showed a decrease in the diameter of the edema (3 x 2 mm), and the nonmammalian meats used as control remained negative ( Figure 2 ). Our patient has been consuming 120 mg of meat daily for about a month without any problems.

**Table 1:** Read meat desensitization protocol

Cooked meat exract: B solution: 1/1000 dilution (0.001 mg/mL), 1 mL= 20 drop			
Days	First Dose	Second Dose	Daily cumulative dose
1	10 drops= 0.5 mL (0.0005 mg)	10 drops= 0.5 mL (0.0005 mg)	20 drops (0.001 mg)
2	10 drops= 0.5 mL (0.0005 mg)	10 drops= 0.5 mL (0.0005 mg)	20 drops (0.001 mg)
3	20 drops= 1 mL (0.001 mg)	20 drops= 1 mL (0.001 mg)	40 drops (0.002 mg)
4	40 drops= 2 mL (0.002 mg)	40 drops= 2 mL (0.002 mg)	80 drops (0.004 mg)
5	50 drops= 2.5 mL (0.003 mg)	50 drops= 2.5 mL (0.003 mg)	100 drops (0.006 mg)
6-8	60 drops= 3 mL (0.004 mg)	60 drops= 3 mL (0.004 mg)	120 drops (0.008 mg)
Cooked meat exract: A solution: 1/100 dilution (0.01 mg/mL), 1 mL= 20 drop			
9	20 drops= 1 mL (0.01 mg)	20 drops= 1 mL (0.01 mg)	40 drops (0.02 mg)
10	40 drops= 2 mL (0.02 mg)	40 drops= 2 mL (0.02 mg)	80 drops (0.04 mg)
11	80 drops= 4 mL (0.04 mg)	80 drops= 4 mL (0.04 mg)	160 drops (0.08 mg)
Cooked Pure Meat			
12	0.1 mg	0.1 mg	0.2 mg
13-15	0.2 mg	0.2 mg	0.4 mg
16	0.5 mg *	0.5 mg *	1 mg
17	1 mg	1 mg	2 mg
18	2 mg	2 mg	4 mg
19	4 mg	4 mg	8 mg
20-22	8 mg	8 mg	16 mg
23	16 mg	16 mg	32 mg
24	32 mg	32 mg	64 mg
25	64 mg	64 mg	128 mg
26	128 mg	128 mg	256 mg
27-29	256 mg	256 mg	512 mg
30	500 mg	500 mg	1 gr
31	1 gr	1 gr	2 gr
32	2 gr	2 gr	4 gr
33	4 gr	4 gr	8 gr
34-36	8 gr	8 gr	16 gr
37	16 gr	16 gr	32 gr
38	32 gr	32 gr	60 gr
39	60 gr	60 gr	120

Solution A: 6 mg meat (0.01 mg/mL) boiled in 600 ml water for 15 minutes Solution B: 10 ml solution A + 90 mL water (0.001 mg/mL) *Local urticaria plaque developed on the anterior abdominal wall at a dose of 40 drops (0.002 mg) switched to 0.5 mg. Light gray area: Intermediate steps were added. Dark gray area: Consantration.

## DISCUSSION

Our patient, whom we successfully desensitized due to Alpha-gal allergy, had risk factors for Alpha-gal allergy due to repeated exposure to tick bites from working in hazelnut fields and having A Rh+ blood group. However, cat sensitization (Fel d2), which can cross-react with the Alpha-gal epitope, was not detected, while honeybee sensitization (similar epitope api m1 positive) was detected. The insects that were positive in the ALEX test were attributed to possible cross-reactivity with ticks. There were no risk factors such as non-steroidal anti-inflammatory drug allergy, alcohol and exercise that could exacerbate the reaction. Positive prick to prick test with cooked and raw beef, positive prick and intradermal test with cetuximab and positive Alpha-gal sIgE (27.3 Ku/L) together with the history led to the diagnosis of Alpha-gal syndrome. Although Alpha-gal allergy was not confirmed by oral provocation in this case, a study reported that Alpha-gal sIgE> 5.5 kU/L indicated red meat allergy with 95% probability ( 21 ). In fact, a mild reaction developed during desensitization in our patient. In addition, oral provocation is recommended in patients in whom history and tests are incompatible ( 18 ).

Oral desensitization protocols developed for induction of oral tolerance in food allergy are promising especially for children with peanut, milk and egg allergy ( 22 ). Although desensitization is not generally recommended in Alpha-gal allergy, which we have started to encounter frequently in adults, many meals in the food culture in our country contain red meat products such as minced meat or broth. The need to prepare meals separately from the family at every meal negatively affected the quality of life of our patient. Therefore, desensitization should be kept in mind in the treatment of these patients in our country. The two desensitization protocols in the literature have also been reported from our country, the first one was started with 1/1000 dilution and the other with 1/100 dilution; the most important deficit we observed in both protocols was an increase of more than 100-fold in the step from meat exract to pure meat ( 10 , 19 ). However, in oral immunotherapy with foods such as milk and peanuts, one and a half-twicefold dose increases have been applied in the induction phase ( 22 , 23 ). Excessive dose increase may increase the possibility of allergic reaction as in our patient. At this point, we ensured a more reliable and successful completion of desensitization with the intermediate steps we introduced. In addition, the decrease in the diameter of edema in the prick to prick test with red meat supports the success of desensitization in a concrete way. Our patient, who started 15 years ago and described a reaction up to anaphylaxis about two hours after meat consumption many times, has safely regained the consumption of red meat that he had been longing for years after the successful desensitization we applied.

## CONFLICT of INTEREST

The authors have no conflict of interest to declare.

## AUTHORSHIP CONTRIBUTIONS

Concept/Design: All of authors

Analysis/Interpretation: FMT, ZYK

Data acqusition: FMT, RBB

Writing: FMT, ZYK

Clinical Revision: BÇU, FMT

Final Approval: All of authors
